# Supplementary material for: Impacts of smoke-free public places legislation on inequalities in youth smoking uptake: study protocol for a secondary analysis of UK survey data
Source: BMJ Open. 2018 Mar 27;8(3):e022490. doi: 10.1136/bmjopen-2018-022490 (PMC5875608; doi:10.1136/bmjopen-2018-022490)
Supplement: Author's manuscript [file bmjopen-2018-022490.draft_revisions.pdf]

BMJ Open is committed to open peer review. As part of this commitment we make the peer review history of every article we publish publicly available.

When an article is published we post the peer reviewers' comments and the authors' responses online. We also post the versions of the paper that were used during peer review. These are the versions that the peer review comments apply to.

The versions of the paper that follow are the versions that were submitted during the peer review process. They are not the versions of record or the final published versions. They should not be cited or distributed as the published version of this manuscript.

BMJ Open is an open access journal and the full, final, typeset and author-corrected version of record of the manuscript is available on our site with no access controls, subscription charges or pay-per-view fees (http://bmjopen.bmj.com).

If you have any questions on BMJ Open's open peer review process please email info.bmjopen@bmj.com

BMJ Open

IMPACTS OF SMOKE-FREE PUBLIC PLACES LEGISLATION ON INEQUALITIES IN YOUTH SMOKING UPTAKE: STUDY PROTOCOL

Journal:	*BMJ Open*
Manuscript ID	bmjopen-2018-022490
Article Type:	Protocol
Date Submitted by the Author:	22-Feb-2018

Complete List of Authors:	Anyanwu, Philip; University of Glasgow, MRC/CSO Social and Public Health Sciences Unit<br>Craig , Peter ; University of Glasgow, MRC/CSO Social and Public Health Sciences Unit<br>Katikireddi, Srinivasa; University of Glasgow, MRC/CSO Social and Public Health Sciences Unit<br>Green, Michael; University of Glasgow, MRC/CSO Social and Public Health Sciences Unit

Keywords:	smoking, youth, inequalities, smoke-free legislation, longitudinal, United Kingdom

SCHOLARONE™ Manuscripts

For peer review only

Title of article: **IMPACTS OF SMOKE-FREE PUBLIC PLACES LEGISLATION ON INEQUALITIES IN YOUTH SMOKING UPTAKE: STUDY PROTOCOL**

Corresponding Author: **Philip Emeka Anyanwu** PhD
MRC/CSO Social and Public Health Sciences Unit
Institute of Health and Well-being
University of Glasgow
200 Renfield Street,
Glasgow
G2 3QB
Email: anyanwuphilipemeka@gmail.com
philip.anyanwu@glasgow.ac.uk
Tel: +44(0)141 353 7527

Co-authors: **Peter Craig** PhD
MRC/CSO Social and Public Health Sciences Unit
Institute of Health and Well-being
University of Glasgow
Glasgow
United Kingdom

Srinivasa Vittal Katikireddi PhD
MRC/CSO Social and Public Health Sciences Unit
Institute of Health and Well-being
University of Glasgow
Glasgow
United Kingdom

Michael James Green PhD
MRC/CSO Social and Public Health Sciences Unit
Institute of Health and Well-being

For peer review only - http://bmjopen.bmj.com/site/about/guidelines.xhtml

University of Glasgow

Glasgow

United Kingdom

Key words: Smoking, Youth, Inequalities, Smoke-Free Legislation, United Kingdom

Word count: 3886

For peer review only

For peer review only - http://bmjopen.bmj.com/site/about/guidelines.xhtml

Abstract

Introduction

Smoke-free public places legislation has been introduced in many countries to protect the public from the harmful effects of second-hand smoking. While evaluations of smoke-free policies have demonstrated major public health benefits, the impact on youth smoking and inequalities in smoking remains unclear. This project aims to evaluate how smoke-free public places legislation in the UK has impacted on inequalities in youth smoking uptake, and how much of any impact is via changes in parental smoking behaviour.

Methods and analysis

The study will constitute secondary analyses of UK data (from the British Household Panel Survey and the Understanding Society study). Merging these datasets gives coverage of the period from 1994 to 2016. Missing data will be handled using multiple imputation. The primary outcomes are the rates and inequalities in initiation, experimentation, escalation to daily smoking, and quitting among youths aged 11 to 15 years. Secondary outcomes include the prevalence of smoking among parents of these youths. Discrete-time event history analysis will be conducted to examine whether changes in the probability of youth smoking transitions are associated with the implementation of the smoke-free public places legislation; and whether any observed effects differ by socioeconomic position and parental smoking. A multi-level logistic regression model will be used to investigate whether there is a step-change or change in trend for the prevalence of parental smoking after the policy was implemented. The models will be adjusted for relevant factors (including cigarette taxation, the change in the legal age for purchase of cigarettes, and e-cigarette prevalence) that may be associated with the implementation of the legislation.

Ethics and dissemination

This project will use anonymised survey data which has been collected following independent ethical review. The dissemination of the study findings will adopt multiple communication channels targeting both scientific and non-scientific audiences.

Registration

Not applicable.

Strengths and limitations of this study

- The study will provide novel evidence on the impact of UK smoke-free public places legislation on inequalities in distinct stages of youth smoking uptake

For peer review only - http://bmjopen.bmj.com/site/about/guidelines.xhtml

- We utilise household data to investigate changes in parental smoking as one potential mechanism by which the legislation may impact on youth smoking uptake (or inequalities therein)
- Causal attribution of observed changes to the smoke-free legislation rests on the assumption that this was the only relevant change occurring at this point in time
- Nevertheless, we plan sensitivity analyses to adjust for other relevant policy changes and strengthen the case that any observed impacts are specifically associated with the smoke-free legislation

Introduction

Tobacco smoking remains a major cause of death and disability around the world, as well as a major contributor to health inequalities (1,2). Socioeconomic inequalities in youth smoking uptake are an important driver of adult inequalities in smoking (3–5), as the habit is difficult to quit once established. Tackling the uptake of smoking amongst adolescents is, therefore, a key aspect of achieving national goals for a smoke-free generation (1,6,7).

Smoke-free public places legislation (8) has been introduced in many countries to protect the public, especially non-smokers, from the harmful effects of second-hand smoking (SHS). Evaluations of smoke-free policy have demonstrated it has had major public health benefits, including reducing rates of heart attack and pregnancy-related complications (8–13). Positive impacts on child health have also been seen, with smoke-free public places policy linked to reductions in childhood asthma hospitalisations (9). However, a recent Cochrane systematic review found sparse and inconsistent evidence as to the impact of such policies on inequalities in smoking prevalence and even sparser evidence regarding the policies' impacts on overall and inequalities in smoking uptake amongst young people (8). One study of smoking prevalence among 13 and 15-year-old adolescents in the UK has suggested a drop in prevalence after legislation, particularly for females; but did not examine socioeconomic inequalities (14).

Although its primary purpose was to prevent harm to the public, especially non-smokers, from SHS, the smoke-free public places legislation in the UK was also expected to improve air quality in public places and reduce health risks associated with smoking amongst smokers (15). Importantly, the legislation was also expected to contribute to changes in social attitudes and norms towards smoking by reducing the acceptability of smoking, especially in social

For peer review only - http://bmjopen.bmj.com/site/about/guidelines.xhtml

places like pubs and restaurants and other public spaces shared with non-smokers (16,17). For example, research on implementation of smoke-free legislation in Massachusetts, US showed that smoking became less socially acceptable, and social norms shifted to favour smoke-free environments (18). Impacts on adult behaviour and societal norms are critical to understanding how and why a ban might impact on youth smoking, as youths are unlikely to smoke in the public places affected by the legislation. Any effect on youth smoking could be through changes in adult smoking behaviour, and the implications this has for social norms and the availability of cigarettes to young people -given that adults are a major source of cigarettes for youth smokers (19,20). Thus, effects of legislation may not be immediate but may grow over the years following the implementation of the legislation as these effects accumulate.

Parental smoking is a key influence on children's smoking uptake (5,21) and thus may be a major mechanism by which smoke-free legislation impacts on youth smoking. There are at least two potential mechanisms: impacts on the prevalence of parental smoking, and displacement of smoking behaviour (see Figure 1). First, regarding the prevalence of parental smoking, while studies tend not to show long-term effects of legislation on overall smoking prevalence, heterogeneity of impact among sub-groups of smokers is still largely unexplored (8,22). Smokers who live with their children are one sub-group among whom smoking legislation and its associated social changes might be expected to have a stronger impact. Social de-normalisation of smoking via smoke-free legislation may raise adults' consciousness and awareness of potential harms, especially around children and non-smokers (23). Concerns about the health of children may then provoke cessation attempts. If parental smoking reduces after a ban, then decreases in youth uptake might be expected to follow.

Another potential, and not mutually-exclusive, impact of smoke-free legislation is displacement of parental smoking behaviour (i.e. not whether but where parents smoke). The displacement could be into or out of less regulated environments such as the home (24,25), which could influence children's exposure to smoking behaviour. Indeed, biomarker data from one US study has indicated increased exposure to cigarette smoke among children living with smokers after public smoking bans (26). Nevertheless, evidence for displacement into the home is inconsistent: several studies have shown reductions rather than increases in the prevalence of smoking at home after smoke-free legislation (27,28) and more voluntary smoke-free homes (23,29–36), especially homes with youths aged 15 years or less (37). If

For peer review only - http://bmjopen.bmj.com/site/about/guidelines.xhtml

smoking is displaced out of the home, this may both reduce youths' exposure to SHS and potentially reduce the uptake of smoking in children (38) by weakening the influence of parental smoking on youth uptake. Alternatively, if parental smoking were displaced into homes, then the reverse could be true: an increase in uptake due to a strengthening of the influence of parental smoking.

Inconsistencies in existing evidence suggest heterogeneity in effects of the smoke-free public places legislation, so it is vital to explore the effects on inequalities. Considering the likely importance of parental smoking in any impact of legislation on youth uptake and that young people from disadvantaged backgrounds are more likely to have parents who smoke, it is likely that inequalities in youth uptake will be affected. Particularly, if more advantaged smoking parents are more likely to quit or stop smoking at home after a ban, or if disadvantaged smoking parents are more likely to displace their smoking behaviour into the home, then inequalities in youth uptake might be expected to widen. Indeed, initial data using salivary cotinine samples from school children before and after implementation of UK bans do suggest a widening of inequalities in children's exposure to smoking, with greater decreases in exposure among the more affluent (39,40).

Randomised controlled trials, often seen as the gold standard for establishing causality, are ethically and practically problematic in the context of studying population-level policies such as the smoke-free legislation. However, implementation of smoke-free public places legislation in the UK, which occurred in 2006 in Scotland and 2007 in the rest of the UK, provides the opportunity for a natural experimental approach (41) to studying the impact of this policy on youth uptake, and the mechanisms by which these impacts occur. A natural experimental approach requires weaker assumptions for observed effects to be interpreted as causal than in most observational epidemiological studies. Specifically, if we were to observe that both parental smoking and the implementation of smoke-free legislation were associated with youth smoking uptake (thinking of parental smoking generally rather than in the context of this policy change), then causal interpretations of these associations both require an assumption of no (unmeasured) confounding. However, this assumption is considerably stronger for parental smoking (i.e. that no other factor is causing both parental smoking and youth smoking) than for the timing of the legislation (i.e. that no other factor causing/coinciding with the implementation of the legislation implementation also causes changes in youth smoking uptake).

For peer review only - http://bmjopen.bmj.com/site/about/guidelines.xhtml

Nevertheless, even for the implementation of smoke-free legislation, the assumption of no confounding may be problematic as the policy has not been the only important change happening in the UK that might impact on youth smoking uptake. Though acknowledged as one of the most significant changes in policy, smoke-free legislation was implemented in the UK within a period of incrementally increasing tobacco control (42). For example, sales taxes applied to cigarettes are subject to ongoing change, and the implementation of smoke-free legislation also coincided closely with a 2007 change in the legal age for purchase of cigarettes from 16 to 18 years. Another factor is the availability and use of e-cigarettes which has been growing in the UK since 2011 (43). To attribute changes in youth smoking to the smoke-free policies, we will need to differentiate the impacts of the policy from these other contextual changes.

Aims/Objectives/Research questions

This project aims to evaluate how the implementation of the smoke-free public places legislation in the UK has impacted on inequalities in youth smoking uptake. Further, we will investigate how much (if any) of the impact is due to impacts of the ban on the smoking behaviour of youths' parents. We will seek to differentiate the effects of the smoke-free public places legislation from other changes happening in the UK such as increases in the legal age for purchasing cigarettes, the rising prevalence of e-cigarettes, and the changes in tobacco taxation in the UK.

To ensure this study covers appropriate ages with regards to smoking uptake, we will focus on youths aged 11-15 years. We focus on smoking uptake within this age group because young people who establish a daily smoking habit by age 15 are less likely to quit or reduce their smoking as they move into adulthood (21,44–46). Smoking uptake in this study is defined as a series of transitions: initiation, representing initial trying; experimentation, or progression from initial trying to occasional use; escalation from occasional to daily use; and quitting (see Figure 2 (47)).

Using population representative longitudinal survey data, we will address the following research questions:

Q1. Has the implementation of the smoke-free public places legislation in the UK been associated with a step-change or change in the trend in the probability of youth smoking transitions?

For peer review only - http://bmjopen.bmj.com/site/about/guidelines.xhtml

Q2. Has the implementation of the smoke-free public places legislation in the UK been associated with a step-change or change in the trend in the inequalities in the probability of youth smoking transitions?

Q3 (a) Has the implementation of the smoke-free public places legislation in the UK been associated with a step-change or change in the trend in the prevalence of smoking among parents of UK youths? (b) Does the observed change (if any) differ by socioeconomic position?

Q4. (a) Do changes in parental smoking explain any impact of the smoke-free public places legislation on the probability of youth smoking transitions? (b) Has the implementation of the smoke-free public places legislation in the UK been associated with any change in the strength of the association between parental smoking and the probability of youth transitions? (c) Does the observed change (if any) differ by socioeconomic position?

Q5. How distinct are the impacts of the smoke-free public places legislation from the impacts of the change in the legal age for purchasing tobacco, the increasing prevalence of e-cigarettes, and the changes in tobacco taxation on youth smoking transition?

Methods and Analysis

Study design

The study will constitute secondary analyses of UK survey data (see below). The control group will be all UK youths aged 11-15 prior to the implementation of the smoking ban (in 2006 for youths living in Scotland and 2007 for those living elsewhere in the UK). The intervention group is all UK youths aged 11-15 after the implementation of the ban. The primary outcomes of interest are the rates of initiation, experimentation, escalation to daily smoking, and quitting within this age group. A secondary outcome of interest is the prevalence of smoking among parents of these youths. The causal interpretation of the differences between these groups rests on the assumption that the implementation of the smoke-free public places legislation is the only relevant difference between these groups, and that there are no other systematic differences between these groups that would account for their differences in outcomes.

Data

For peer review only - http://bmjopen.bmj.com/site/about/guidelines.xhtml

Data from the British Household Panel Survey Youth Sub-Sample and Understanding Society, both of which are freely available to *bona fide* researchers from the UK Data Archive under their normal terms and conditions (48,49). Both Understanding Society and BHPS studies obtained consent for data sharing from their participants. More information on the data sources, consent, and sampling can be found on BHPS and Understanding Society websites https://www.iser.essex.ac.uk/bhps and https://www.understandingsociety.ac.uk/ respectively. Data on youths' (11-15-year-olds) and parents' smoking history, and parents' socioeconomic position from the British Household Panel Survey (covering the period 1994 to 2009) will be merged with those of the recent Understanding Society Survey (from 2009 to 2016). The merger of these datasets gives coverage of the period from 1994 to 2016, with data from 2016 newly released in November 2017. As such, nine to ten years of post-legislation data will be available in the dataset. This amount of post-legislation data will be important in investigating the immediate as well as the long-term impact of the smoke-free public places legislation on youths smoking transition. Respondents will be included for each year they were aged 11-15 within this period (i.e. a maximum of five years of data per respondent).

Handling missing data

Missing data will be handled using multiple imputation, which ensures that all observed values in the set are retained (50,51). The multiple imputation will be conducted using an unconstrained two-level model in Mplus 8 (52), with person-years nested within persons.

Statistical analysis

Discrete-time event history analysis (53) will be conducted to examine whether changes in the probability of youth smoking transitions are associated with the implementation of the smoke-free public places legislation (Q1). The implementation of smoke-free legislation is viewed as a natural experiment (41,54), and analyses will examine if there is a step change or change in trend in the probability of smoking transitions after the country-specific implementation dates for the smoke-free legislation (i.e. 2006 for Scotland, 2007 for the rest of the UK). Youth will only be considered at risk for a transition once they have made the previous transition (i.e. they are only at risk for experimentation once they have tried smoking). Thus, data for each analysis will be right-censored at the year of the smoking transition (or age 15) and left censored prior to the previous transition (or age 11). Escalation

For peer review only - http://bmjopen.bmj.com/site/about/guidelines.xhtml

to daily smoking and quitting will be treated as two alternative outcomes, which young people will be at risk of once they commence occasional smoking. In line with previous work (47), youths who skipped smoking transition stages will be treated as making the intervening transitions in the same year. For instance, youths who transited from never having tried smoking to ex-smoker in the same year will be treated as having progressed to occasional smoker and having quit within the same year (but not as having escalated to daily smoking). Analyses will be adjusted for age, gender, the overall temporal trend in rates (which may encompass effects of other temporal increases in tobacco control), and overall differences in rates between UK countries.

We will then examine whether any effects observed on youth smoking transition differ by socioeconomic position (Q2). Parental education level will be used as an indicator of socioeconomic position. In cases where parental educational qualification for both parents differs, the higher qualification will be used. This will be added to the predictive models, alongside interactions with the variables representing the implementation of the legislation.

A multi-level logistic regression model (with person-years nested within persons) will be used to investigate whether there is a step-change or change in trend for the prevalence of parental smoking (any current smoking by either parent) after smoke-free public places legislations were implemented in the UK (Q3a); and whether observed changes (if any) differ by socioeconomic position (Q3b). This model will include all years of data for all respondents without any right or left censoring and will be adjusted for the young person's age, gender, the overall temporal trend in parental smoking, and overall differences between UK countries. The second stage of the modelling will additionally include parental education and interactions between parental education and the variables representing the implementation of the legislation.

Next, building on the models developed to assess whether the impact of the legislation on youth smoking transitions varied by socioeconomic position (Q2), parental smoking will also be included and we will assess whether this explains any observed differences associated with the implementation of the legislation (Q4a). We will test for interactions between parental smoking and the variables representing the implementation of the legislation to see if the influence of parental smoking changes after the legislation (Q4b), and then for a three-way interaction between parental smoking, legislation implementation and socioeconomic position to see if this varies by socioeconomic position (Q4c).

For peer review only - http://bmjopen.bmj.com/site/about/guidelines.xhtml

A final step will be to adjust the above models for relevant factors that may be associated with the implementation of the legislation. This will include annual levels of cigarette taxation, the change in the legal age of purchase for cigarettes from 16 to 18 in 2007 (that is, the implementation date differs from that of the smoke-free legislation by one year in Scotland only), and survey estimates of the annual prevalence of e-cigarettes from 2012 (55) (with e-cigarette prevalence coded as 0 prior to that date). As an additional sensitivity test, we will re-run analyses with the date of ban implementation transposed either 5 years forward or backward in time. If observed effects are genuinely attached to the ban rather than overall temporal trends, these transposed 'placebo' effect estimates should be weaker or null compared to those observed at the actual ban implementation dates.

Patient and Public Involvement

This study will not involve patients. The study will constitute secondary analyses of UK survey data. The dissemination of the results will include communication channels and public engagement events that will involve youths and parents.

Beneficiaries and target audiences

The project will be of interest to a range of academic and non-academic audiences. Research on tobacco control has a broad audience including epidemiologists, public health, policy researchers, governmental organisations, and the UK general public. The findings of this research will be of benefit to the governments of UK countries as they will provide information on important impacts of an existing policy. This information will be of use if the policy is ever considered for repeal or modification. The evidence on the mechanisms of impact on young people's smoking transition via their parents that will be generated from this project will be particularly valuable when considering extensions to smoke-free policies (for example, to more private spheres such as cars and homes, or to other public spaces such as outdoor parks) or other tobacco control policies which may have impacts on youth via parental smoking.

Another group who may benefit from this study are policymakers in other countries. Findings will be of international relevance as many countries are yet to implement partial or complete smoke-free public places legislation (56). The understanding of impacts and mechanisms gained from this study will help international policymakers evaluate whether similar effects could be gained by implementing smoke-free or other policies within their own countries.

For peer review only - http://bmjopen.bmj.com/site/about/guidelines.xhtml

Limitations

The causal interpretation of any impact on youth smoking uptake or parental smoking that we observe to be associated with the implementation of the smoke-free legislation rests on the assumption that this is the only relevant difference occurring at that point in time. Nevertheless, we will adjust for effects of some other relevant differences that could bias the results such as changes in tobacco taxation and the increasing availability of e-cigarettes, to see if we can differentiate the effects of the smoke-free legislation. The change in the legal age for purchase of cigarettes from age 16 to 18 in 2007 is particularly problematic, as it coincides so closely with the timing of the implementation of the smoke-free legislation (only differing by a year in Scotland, and being concurrent in the rest of the UK), so it may be difficult to statistically distinguish their effects. At worst this means that findings could be interpreted in terms of the package of policies implemented at that point (i.e. the smoke-free legislation and the change in legal age), but our investigation of mechanisms of impact via parental smoking remains relevant in this regard. If some of the impacts can be attributed to parental smoking, this will further strengthen the case for a causal effect of the smoke-free legislation, as the change in legal age could be expected to have much less of an impact on adult smokers.

Discussion

Smoke-free public places legislation has already been the subject of many evaluative studies and is widely viewed as a success, particularly in reducing exposure to second-hand smoke; but there remain gaps in the evidence base for this policy. This project would fill one of the most important of these gaps, relating to a crucial population group in tobacco control, i.e. youth. If the project shows positive impacts of the smoke-free legislation, then it will add to the evidence supporting this policy. If the project shows negative impacts of the smoking ban -such as the widening of inequalities in youth smoking uptake-, then it provides important information that should be weighed against the other benefits already shown in previous research; and may point to other measures that might be taken in combination with smoke-free legislation for maximum benefit.

The difficulty in establishing whether observed associations between events are causal is a common problem in the field of epidemiology (57), but our natural experimental approach will help strengthen the case for a causal effect. Furthermore, the interest of evaluators of

For peer review only - http://bmjopen.bmj.com/site/about/guidelines.xhtml

social policies or interventions goes beyond overall effects. An understanding of the mechanisms by which an effect occurs is crucial to social policy evaluators. Such understanding can contribute to establishing the transferability of such policies to different contexts while achieving similar effects. This project will achieve this grounded understanding by explicating the role of parental smoking in any impact on youth smoking transition. Similarly, the fine definition of smoking transition which includes all stages of smoking (from initiation to escalation or quitting) will help elucidate which specific stages of the process of smoking transition are affected by the smoke-free public places legislation, and to what extent the effect is.

Finally, e-cigarettes are the focus of current controversy in the field of tobacco control. Many advocate e-cigarettes for the potential harm-reduction which could be achieved if smokers switched from tobacco to e-cigarettes, but there are also concerns that they may re-normalise smoking behaviours (58), and put current successes in tobacco control at risk. A particular concern is that e-cigarettes could help establish nicotine addiction in young people and lead to increases in cigarette smoking (59), though there is little evidence of this as yet (43). By differentiating effects of the smoke-free public places legislation from those of increasing e-cigarette prevalence, we will generate evidence about the independent effects of each on youth smoking transitions, making an important contribution to these current policy debates.

Ethical and dissemination

This study will utilise secondary data that are anonymised and obtained from studies that have already undergone ethical review. The Understanding Society study protocol was reviewed and approved by the Ethics Committee of the University of Essex; while the BHPS complied with the ethical guidelines of the Social Research Association.

The dissemination of the study findings will adopt multiple communication channels targeting both scientific and non-scientific audiences. The key communication channels will include peer-reviewed journal articles, conference presentations, press releases coinciding with publications, online blogs, and public engagement events. Also, policymakers and stakeholders will be updated on the progress of this study and on preliminary findings via a virtual stakeholder network.

For peer review only - http://bmjopen.bmj.com/site/about/guidelines.xhtml

References

1. Ali R, Hay S. Smoking prevalence and attributable disease burden in 195 countries and territories, 1990–2015: a systematic analysis from the Global Burden of Disease Study 2015. 2017;

2. Forouzanfar MH, Afshin A, Alexander LT, Aasvang GM, Bjertness E, Htet AS, et al. Global, regional, and national comparative risk assessment of 79 behavioural, environmental and occupational, and metabolic risks or clusters of risks, 1990-2015: a systematic analysis for the Global Burden of Disease Study 2015. 2016;

3. Brown T, Platt S, Amos A. Equity impact of interventions and policies to reduce smoking in youth: systematic review. Tob Control. 2014;tobaccocontrol–2013.

4. Hiscock R, Bauld L, Amos A, Fidler JA, Munafò M. Socioeconomic status and smoking: a review. Ann N Y Acad Sci. 2012;1248(1):107–123.

5. Barreto SM, de Figueiredo RC, Giatti L. Socioeconomic inequalities in youth smoking in Brazil. BMJ Open. 2013;3(12):e003538.

6. Global and Public Health/Population Health/HB/ cost centre. Towards a smoke-free generation: tobacco control plan for England - GOV.UK [Internet]. 2017 [cited 2017 Oct 25]. Available from: https://www.gov.uk/government/publications/towards-a-smoke-free-generation-tobacco-control-plan-for-england

7. The Scottish Government. Creating a Tobacco-Free Generation: A Tobacco Control Strategy for Scotland - Google Search [Internet]. The Scottish Government, Edinburgh; 2013 [cited 2017 Oct 25]. Available from: http://www.gov.scot/resource/0041/00417331.pdf

8. Frazer K, Callinan JE, McHugh J, van Baarsel S, Clarke A, Doherty K, et al. Legislative smoking bans for reducing harms from secondhand smoke exposure, smoking prevalence and tobacco consumption. Cochrane Libr [Internet]. 2016 [cited 2017 Sep 18]; Available from: http://onlinelibrary.wiley.com/doi/10.1002/14651858.CD005992.pub3/full

9. Mackay D, Haw S, Ayres JG, Fischbacher C, Pell JP. Smoke-free legislation and hospitalizations for childhood asthma. N Engl J Med. 2010;363(12):1139–1145.

10. Pell JP, Haw S, Cobbe S, Newby DE, Pell AC, Fischbacher C, et al. Smoke-free legislation and hospitalizations for acute coronary syndrome. N Engl J Med. 2008;359(5):482–491.

11. Mackay DF, Nelson SM, Haw SJ, Pell JP. Impact of Scotland's smoke-free legislation on pregnancy complications: retrospective cohort study. PLoS Med. 2012;9(3):e1001175.

12. Been JV, Nurmatov UB, Cox B, Nawrot TS, van Schayck CP, Sheikh A. Effect of smoke-free legislation on perinatal and child health: a systematic review and meta-analysis. The Lancet. 2014;383(9928):1549–1560.

For peer review only

For peer review only - http://bmjopen.bmj.com/site/about/guidelines.xhtml

13. Faber T, Kumar A, Mackenbach JP, Millett C, Basu S, Sheikh A, et al. Effect of tobacco control policies on perinatal and child health: a systematic review and meta-analysis. Lancet Public Health. 2017;2(9):e420–e437.

14. Katikireddi SV, Der G, Roberts C, Haw S. Has childhood smoking reduced following smoke-free public places legislation? A segmented regression analysis of cross-sectional UK school-based surveys. Nicotine Tob Res. 2016;18(7):1670–1674.

15. Britton J. Smoke-free policy and child health. Lancet Public Health. 2017;2(9):e392–e393.

16. Ritchie D, Amos A, Martin C. Public places after smoke-free—A qualitative exploration of the changes in smoking behaviour. Health Place. 2010 May 1;16(3):461–9.

17. Haw SJ, Gruer L, Amos A, Currie C, Fischbacher C, Fong GT, et al. Legislation on smoking in enclosed public places in Scotland: how will we evaluate the impact? J Public Health. 2006;28(1):24–30.

18. Hamilton WL, Biener L, Brennan RT. Do local tobacco regulations influence perceived smoking norms? Evidence from adult and youth surveys in Massachusetts. Health Educ Res. 2007;23(4):709–722.

19. Lenk KM, Toomey TL, Shi Q, Erickson DJ, Forster JL. Do sources of cigarettes among adolescents vary by age over time? J Child Adolesc Subst Abuse. 2014;23(2):137–143.

20. Jansen P, Toomey TL, Nelson TF, Fabian LE, Lenk KM, Forster JL. Sources of cigarettes among adolescent smokers: Free or purchased? Am J Health Educ. 2011;42(3):154–160.

21. Tyas SL, Pederson LL. Psychosocial factors related to adolescent smoking: a critical review of the literature. Tob Control. 1998;7(4):409–420.

22. Jones AM, Laporte A, Rice N, Zucchelli E. Do public smoking bans have an impact on active smoking? Evidence from the UK. Health Econ. 2015;24(2):175–192.

23. Monson E, Arsenault N. Effects of enactment of legislative (public) smoking bans on voluntary home smoking restrictions: A review. Nicotine Tob Res. 2017;19(2):141–148.

24. House of Commons - Health - Minutes of Evidence. Examination of witnesses (questions 1–19) 23rd Feb 2005 [Internet]. 2005 [cited 2017 Sep 18]. Available from: https://publications.parliament.uk/pa/cm200405/cmselect/cmhealth/358/5022302.htm

25. López MJ, Nebot M, Schiaffino A, Pérez-Ríos M, Fu M, Ariza C, et al. Two-year impact of the Spanish smoking law on exposure to secondhand smoke: evidence of the failure of the 'Spanish model.' Tob Control. 2011;tc–2010.

26. Adda J, Cornaglia F. The effect of bans and taxes on passive smoking. Am Econ J Appl Econ. 2010;2(1):1–32.

27. Lee JT, Glantz SA, Millett C. Effect of smoke-free legislation on adult smoking behaviour in England in the 18 months following implementation. PloS One. 2011;6(6):e20933.

For peer review only - http://bmjopen.bmj.com/site/about/guidelines.xhtml

28. Carpenter C, Postolek S, Warman C. Public-place smoking laws and exposure to environmental tobacco smoke (ETS). Am Econ J Econ Policy. 2011;3(3):35–61.

29. Mons U, Nagelhout GE, Allwright S, Guignard R, van den Putte B, Willemsen MC, et al. Impact of national smoke-free legislation on home smoking bans: findings from the International Tobacco Control Policy Evaluation Project Europe Surveys. Tob Control. 2012;tobaccocontrol–2011.

30. Martínez-Sánchez JM, Blanch C, Fu M, Gallus S, La Vecchia C, Fernández E. Do smoke-free policies in work and public places increase smoking in private venues? Tob Control. 2013;tobaccocontrol–2012.

31. Ferketich AK, Lugo A, La Vecchia C, Fernandez E, Boffetta P, Clancy L, et al. Relation between national-level tobacco control policies and individual-level voluntary home smoking bans in Europe. Tob Control. 2016;25(1):60–65.

32. Hyland A, Hassan LM, Higbee C, Boudreau C, Fong GT, Borland R, et al. The impact of smokefree legislation in Scotland: results from the Scottish ITC Scotland/UK longitudinal surveys. Eur J Public Health. 2009;19(2):198–205.

33. Holliday JC, Moore GF, Moore LA. Changes in child exposure to secondhand smoke after implementation of smoke-free legislation in Wales: a repeated cross-sectional study. BMC Public Health. 2009;9(1):430.

34. Akhtar PC, Currie DB, Currie CE, Haw SJ. Changes in child exposure to environmental tobacco smoke (CHETS) study after implementation of smoke-free legislation in Scotland: national cross sectional survey. Bmj. 2007;335(7619):545.

35. Sims M, Bauld L, Gilmore A. England’s legislation on smoking in indoor public places and work-places: impact on the most exposed children. Addiction. 2012;107(11):2009–2016.

36. Jarvis MJ, Sims M, Gilmore A, Mindell J. Impact of smoke-free legislation on children’s exposure to secondhand smoke: cotinine data from the Health Survey for England. Tob Control. 2011;tc–2010.

37. Lidón-Moyano C, Martínez-Sánchez JM, Fu M, Ballbè M, Martín-Sánchez JC, Martínez C, et al. Secondhand smoke risk perception and smoke-free rules in homes: a cross-sectional study in Barcelona (Spain). BMJ Open. 2017;7(1):e014207.

38. Klein EG, Forster JL, Erickson DJ, Lytle LA, Schillo B. The relationship between local clean indoor air policies and smoking behaviours in Minnesota youth. Tob Control. 2009 Apr 1;18(2):132–7.

39. Moore GF, Currie D, Gilmore G, Holliday JC, Moore L. Socioeconomic inequalities in childhood exposure to secondhand smoke before and after smoke-free legislation in three UK countries. J Public Health. 2012;34(4):599–608.

40. Akhtar PC, Haw SJ, Levin KA, Currie DB, Zachary R, Currie CE. Socioeconomic differences in second-hand smoke exposure among children in Scotland after introduction of the smoke-free legislation. J Epidemiol Community Health. 2010;64(4):341–346.

For peer review only - http://bmjopen.bmj.com/site/about/guidelines.xhtml

41. Craig P, Katikireddi SV, Leyland A, Popham F. Natural Experiments: An Overview of Methods, Approaches, and Contributions to Public Health Intervention Research. Annu Rev Public Health. 2017;38:39–56.

42. Sims M, Salway R, Langley T, Lewis S, McNeill A, Szatkowski L, et al. Effectiveness of tobacco control television advertising in changing tobacco use in England: a population-based cross-sectional study. Addiction. 2014;109(6):986–994.

43. McNeill A, Brose LS, Calder R, Hitchman SC, Hajek P, McRobbie H. E-cigarettes: an evidence update. Public Health Engl. 2015;3.

44. Chassin L, Presson CC, Pitts SC, Sherman SJ. The natural history of cigarette smoking from adolescence to adulthood in a midwestern community sample: multiple trajectories and their psychosocial correlates. Health Psychol. 2000;19(3):223.

45. Kandel DB, Logan JA. Patterns of drug use from adolescence to young adulthood: I. Periods of risk for initiation, continued use, and discontinuation. Am J Public Health. 1984;74(7):660–666.

46. Wellman RJ, DiFranza JR, Savageau JA, Dussault GF. Short term patterns of early smoking acquisition. Tob Control. 2004;13(3):251–257.

47. Green MJ, Leyland AH, Sweeting H, Benzeval M. Socioeconomic position and early adolescent smoking development: evidence from the British Youth Panel Survey (1994–2008). Tob Control. 2016;25(2):203–210.

48. Taylor M, Change ERC on M-S. British Household Panel Survey user manual vol. A: Introduction, technical report and appendices. ESRC Research Centre on Micro-Social Change; 1995.

49. Knies G. User guide – Understanding Society: The UK Household Longitudinal Study waves 1-6 [Internet]. 2016 [cited 2017 Nov 16]. Available from: https://www.understandingsociety.ac.uk/documentation/mainstage

50. Sterne JA, White IR, Carlin JB, Spratt M, Royston P, Kenward MG, et al. Multiple imputation for missing data in epidemiological and clinical research: potential and pitfalls. Bmj. 2009;338:b2393.

51. Allison PD. Missing data: Quantitative applications in the social sciences. Br J Math Stat Psychol. 2002;55(1):193–196.

52. Muthén LK, Muthén BO. Mplus User's Guide. Los Angeles, CA: Muthen & Muthen; 1998-2010. CzeglédiE Body Dissatisfaction Trait Anxiety Self-Esteem Young Men. 2016;

53. Allison PD. Discrete-Time Methods for the Analysis of Event Histories. Sociol Methodol. 1982;13:61–98.

54. Craig P, Cooper C, Gunnell D, Haw S, Lawson K, Macintyre S, et al. Using natural experiments to evaluate population health interventions: new Medical Research Council guidance. J Epidemiol Community Health. 2012;jech–2011.

For peer review only - http://bmjopen.bmj.com/site/about/guidelines.xhtml

55. Action on Smoking and Health. Use of electronic cigarettes (vapourisers) among adults in Great Britain [Internet]. 2016 [cited 2017 Nov 22]. Available from: http://colinmendelsohn.com.au/files/2814/9446/2312/ASH._Use_of_electronic_cigarettes_vapourisers_among_adults_in_Great_Britain._Fact_sheet._May_2017.pdf

56. World Health Organization. WHO REPORT ON THE GLOBAL TOBACCO EPIDEMIC, 2017: Monitoring tobacco use and prevention policies [Internet]. Geneva: WHO; 2017. Available from: http://apps.who.int/iris/bitstream/10665/255874/1/9789241512824-eng.pdf?ua=1&ua=1

57. Goodman S, Samet J. In Schottenfeld, D., and Fraumeni, J. F., (2006) Cancer Epidemiology and Prevention. Oxford University Press; 2006. 1411 p.

58. Fairchild AL, Bayer R, Colgrove J. The renormalization of smoking? E-cigarettes and the tobacco "endgame." N Engl J Med. 2014;370(4):293–295.

59. Hilton S, Weishaar H, Sweeting H, Trevisan F, Katikireddi SV. E-cigarettes, a safer alternative for teenagers? A UK focus group study of teenagers' views. BMJ Open. 2016;6(11):e013271.

Authors' Contributions:

MG, PC and SVK contributed to the conception and design of the study. All authors contributed to developing the analytical methods. The manuscript was drafted by PEA and further revised by MG, PC, SVK and PEA. All authors approved the publication of the protocol.

Funding statement

This study is funded by the Economics & Social Research Council Secondary Data Analysis Initiative (300405-01). All authors receive funding from the Medical Research Council (MC_UU_12017/13 & MC_UU_12017/15) and Scottish Government Chief Scientist Office (SPSHU13 & SPHSU15). SVK is funded by an NRS Senior Clinical Fellowship (SCAF/15/02).

Competing interest

All authors declare no conflicts of interest.

Figure Legends

Figure 1: Conceptual framework for the impact of smoke-free public places legislation on youth smoking transition.

For peer review only - http://bmjopen.bmj.com/site/about/guidelines.xhtml

Figure 2: Smoking stages for early adolescents adapted from ‘Socioeconomic position and early adolescent smoking development: evidence from the British Youth Panel Survey (1994–2008)’ by Green MJ, Leyland AH, Sweeting H, and Benzeval M., 2016, Tobacco Control, 25(2):203–210.

For peer review only

For peer review only - http://bmjopen.bmj.com/site/about/guidelines.xhtml

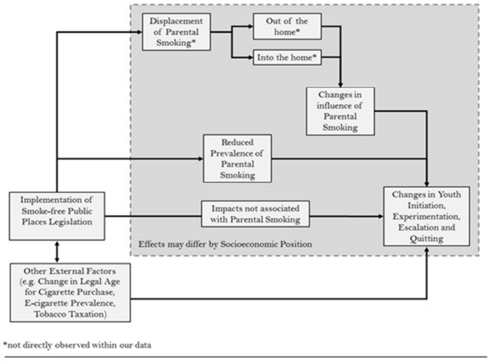

Conceptual framework for the impact of smoke-free public places legislation on youth smoking transition

304x171mm (96 x 96 DPI)

review only

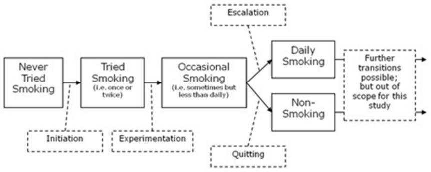

Smoking stages for early adolescents adapted from ‘Socioeconomic position and early adolescent smoking development: evidence from the British Youth Panel Survey (1994–2008)’ by Green MJ, Leyland AH, Sweeting H, and Benzeval M., 2016, Tobacco Control, 25(2):203–210

304x171mm (96 x 96 DPI)

BMJ Open

IMPACTS OF SMOKE-FREE PUBLIC PLACES LEGISLATION ON INEQUALITIES IN YOUTH SMOKING UPTAKE: STUDY PROTOCOL

Journal:	*BMJ Open*
Manuscript ID	bmjopen-2018-022490.R1
Article Type:	Protocol
Date Submitted by the Author:	28-Feb-2018
Complete List of Authors:	Anyanwu, Philip; University of Glasgow, MRC/CSO Social and Public Health Sciences Unit<br>Craig , Peter ; University of Glasgow, MRC/CSO Social and Public Health Sciences Unit<br>Katikireddi, Srinivasa; University of Glasgow, MRC/CSO Social and Public Health Sciences Unit<br>Green, Michael; University of Glasgow, MRC/CSO Social and Public Health Sciences Unit
**Primary Subject Heading**:	Smoking and tobacco
Secondary Subject Heading:	Health policy, Public health, Epidemiology
Keywords:	smoking, youth, inequalities, smoke-free legislation, longitudinal, United Kingdom

SCHOLARONE™ Manuscripts

Title of article: **IMPACTS OF SMOKE-FREE PUBLIC PLACES LEGISLATION ON INEQUALITIES IN YOUTH SMOKING UPTAKE: STUDY PROTOCOL**

Corresponding Author: **Philip Emeka Anyanwu** PhD
MRC/CSO Social and Public Health Sciences Unit
Institute of Health and Well-being
University of Glasgow
200 Renfield Street,
Glasgow
G2 3QB
Email: anyanwuphilipemeka@gmail.com
philip.anyanwu@glasgow.ac.uk
Tel: +44(0)141 353 7527

Co-authors: **Peter Craig** PhD
MRC/CSO Social and Public Health Sciences Unit
Institute of Health and Well-being
University of Glasgow
Glasgow
United Kingdom

Srinivasa Vittal Katikireddi PhD
MRC/CSO Social and Public Health Sciences Unit
Institute of Health and Well-being
University of Glasgow
Glasgow
United Kingdom

Michael James Green PhD
MRC/CSO Social and Public Health Sciences Unit
Institute of Health and Well-being

For peer review only - http://bmjopen.bmj.com/site/about/guidelines.xhtml

University of Glasgow

Glasgow

United Kingdom

Key words: Smoking, Youth, Inequalities, Smoke-Free Legislation, United Kingdom

Word count: 3886

For peer review only

For peer review only - http://bmjopen.bmj.com/site/about/guidelines.xhtml

Abstract

Introduction

Smoke-free public places legislation has been introduced in many countries to protect the public from the harmful effects of second-hand smoking. While evaluations of smoke-free policies have demonstrated major public health benefits, the impact on youth smoking and inequalities in smoking remains unclear. This project aims to evaluate how smoke-free public places legislation in the UK has impacted on inequalities in youth smoking uptake, and how much of any impact is via changes in parental smoking behaviour.

Methods and analysis

The study will constitute secondary analyses of UK data (from the British Household Panel Survey and the Understanding Society study). Merging these datasets gives coverage of the period from 1994 to 2016. Missing data will be handled using multiple imputation. The primary outcomes are the rates and inequalities in initiation, experimentation, escalation to daily smoking, and quitting among youths aged 11 to 15 years. Secondary outcomes include the prevalence of smoking among parents of these youths. Discrete-time event history analysis will be conducted to examine whether changes in the probability of youth smoking transitions are associated with the implementation of the smoke-free public places legislation; and whether any observed effects differ by socioeconomic position and parental smoking. A multi-level logistic regression model will be used to investigate whether there is a step-change or change in trend for the prevalence of parental smoking after the policy was implemented. The models will be adjusted for relevant factors (including cigarette taxation, the change in the legal age for purchase of cigarettes, and e-cigarette prevalence) that may be associated with the implementation of the legislation.

Ethics and dissemination

This project will use anonymised survey data which has been collected following independent ethical review. The dissemination of the study findings will adopt multiple communication channels targeting both scientific and non-scientific audiences.

Registration

Not applicable.

Strengths and limitations of this study

- The study will provide novel evidence on the impact of UK smoke-free public places legislation on inequalities in distinct stages of youth smoking uptake

For peer review only - http://bmjopen.bmj.com/site/about/guidelines.xhtml

- We utilise household data to investigate changes in parental smoking as one potential mechanism by which the legislation may impact on youth smoking uptake (or inequalities therein)
- Causal attribution of observed changes to the smoke-free legislation rests on the assumption that this was the only relevant change occurring at this point in time
- Nevertheless, we plan sensitivity analyses to adjust for other relevant policy changes and strengthen the case that any observed impacts are specifically associated with the smoke-free legislation

Introduction

Tobacco smoking remains a major cause of death and disability around the world, as well as a major contributor to health inequalities (1,2). Socioeconomic inequalities in youth smoking uptake are an important driver of adult inequalities in smoking (3–5), as the habit is difficult to quit once established. Tackling the uptake of smoking amongst adolescents is, therefore, a key aspect of achieving national goals for a smoke-free generation (1,6,7).

Smoke-free public places legislation (8) has been introduced in many countries to protect the public, especially non-smokers, from the harmful effects of second-hand smoking (SHS). Evaluations of smoke-free policy have demonstrated it has had major public health benefits, including reducing rates of heart attack and pregnancy-related complications (8–13). Positive impacts on child health have also been seen, with smoke-free public places policy linked to reductions in childhood asthma hospitalisations (9). However, a recent Cochrane systematic review found sparse and inconsistent evidence as to the impact of such policies on inequalities in smoking prevalence and even sparser evidence regarding the policies' impacts on overall and inequalities in smoking uptake amongst young people (8). One study of smoking prevalence among 13 and 15-year-old adolescents in the UK has suggested a drop in prevalence after legislation, particularly for females; but did not examine socioeconomic inequalities (14).

Although its primary purpose was to prevent harm to the public, especially non-smokers, from SHS, the smoke-free public places legislation in the UK was also expected to improve air quality in public places and reduce health risks associated with smoking amongst smokers (15). Importantly, the legislation was also expected to contribute to changes in social attitudes and norms towards smoking by reducing the acceptability of smoking, especially in social

For peer review only - http://bmjopen.bmj.com/site/about/guidelines.xhtml

places like pubs and restaurants and other public spaces shared with non-smokers (16,17). For example, research on implementation of smoke-free legislation in Massachusetts, US showed that smoking became less socially acceptable, and social norms shifted to favour smoke-free environments (18). Impacts on adult behaviour and societal norms are critical to understanding how and why a ban might impact on youth smoking, as youths are unlikely to smoke in the public places affected by the legislation. Any effect on youth smoking could be through changes in adult smoking behaviour, and the implications this has for social norms and the availability of cigarettes to young people -given that adults are a major source of cigarettes for youth smokers (19,20). Thus, effects of legislation may not be immediate but may grow over the years following the implementation of the legislation as these effects accumulate.

Parental smoking is a key influence on children's smoking uptake (5,21) and thus may be a major mechanism by which smoke-free legislation impacts on youth smoking. There are at least two potential mechanisms: impacts on the prevalence of parental smoking, and displacement of smoking behaviour (see Figure 1). First, regarding the prevalence of parental smoking, while studies tend not to show long-term effects of legislation on overall smoking prevalence, heterogeneity of impact among sub-groups of smokers is still largely unexplored (8,22). Smokers who live with their children are one sub-group among whom smoking legislation and its associated social changes might be expected to have a stronger impact. Social de-normalisation of smoking via smoke-free legislation may raise adults' consciousness and awareness of potential harms, especially around children and non-smokers (23). Concerns about the health of children may then provoke cessation attempts. If parental smoking reduces after a ban, then decreases in youth uptake might be expected to follow.

Another potential, and not mutually-exclusive, impact of smoke-free legislation is displacement of parental smoking behaviour (i.e. not whether but where parents smoke). The displacement could be into or out of less regulated environments such as the home (24,25), which could influence children's exposure to smoking behaviour. Indeed, biomarker data from one US study has indicated increased exposure to cigarette smoke among children living with smokers after public smoking bans (26). Nevertheless, evidence for displacement into the home is inconsistent: several studies have shown reductions rather than increases in the prevalence of smoking at home after smoke-free legislation (27,28) and more voluntary smoke-free homes (23,29–36), especially homes with youths aged 15 years or less (37). If

For peer review only - http://bmjopen.bmj.com/site/about/guidelines.xhtml

smoking is displaced out of the home, this may both reduce youths' exposure to SHS and potentially reduce the uptake of smoking in children (38) by weakening the influence of parental smoking on youth uptake. Alternatively, if parental smoking were displaced into homes, then the reverse could be true: an increase in uptake due to a strengthening of the influence of parental smoking.

Inconsistencies in existing evidence suggest heterogeneity in effects of the smoke-free public places legislation, so it is vital to explore the effects on inequalities. Considering the likely importance of parental smoking in any impact of legislation on youth uptake and that young people from disadvantaged backgrounds are more likely to have parents who smoke, it is likely that inequalities in youth uptake will be affected. Particularly, if more advantaged smoking parents are more likely to quit or stop smoking at home after a ban, or if disadvantaged smoking parents are more likely to displace their smoking behaviour into the home, then inequalities in youth uptake might be expected to widen. Indeed, initial data using salivary cotinine samples from school children before and after implementation of UK bans do suggest a widening of inequalities in children's exposure to smoking, with greater decreases in exposure among the more affluent (39,40).

Randomised controlled trials, often seen as the gold standard for establishing causality, are ethically and practically problematic in the context of studying population-level policies such as the smoke-free legislation. However, implementation of smoke-free public places legislation in the UK, which occurred in 2006 in Scotland and 2007 in the rest of the UK, provides the opportunity for a natural experimental approach (41) to studying the impact of this policy on youth uptake, and the mechanisms by which these impacts occur. A natural experimental approach requires weaker assumptions for observed effects to be interpreted as causal than in most observational epidemiological studies. Specifically, if we were to observe that both parental smoking and the implementation of smoke-free legislation were associated with youth smoking uptake (thinking of parental smoking generally rather than in the context of this policy change), then causal interpretations of these associations both require an assumption of no (unmeasured) confounding. However, this assumption is considerably stronger for parental smoking (i.e. that no other factor is causing both parental smoking and youth smoking) than for the timing of the legislation (i.e. that no other factor causing/coinciding with the implementation of the legislation implementation also causes changes in youth smoking uptake).

For peer review only - http://bmjopen.bmj.com/site/about/guidelines.xhtml

Nevertheless, even for the implementation of smoke-free legislation, the assumption of no confounding may be problematic as the policy has not been the only important change happening in the UK that might impact on youth smoking uptake. Though acknowledged as one of the most significant changes in policy, smoke-free legislation was implemented in the UK within a period of incrementally increasing tobacco control (42). For example, sales taxes applied to cigarettes are subject to ongoing change, and the implementation of smoke-free legislation also coincided closely with a 2007 change in the legal age for purchase of cigarettes from 16 to 18 years. Another factor is the availability and use of e-cigarettes which has been growing in the UK since 2011 (43). To attribute changes in youth smoking to the smoke-free policies, we will need to differentiate the impacts of the policy from these other contextual changes.

Aims/Objectives/Research questions

This project aims to evaluate how the implementation of the smoke-free public places legislation in the UK has impacted on inequalities in youth smoking uptake. Further, we will investigate how much (if any) of the impact is due to impacts of the ban on the smoking behaviour of youths' parents. We will seek to differentiate the effects of the smoke-free public places legislation from other changes happening in the UK such as increases in the legal age for purchasing cigarettes, the rising prevalence of e-cigarettes, and the changes in tobacco taxation in the UK.

To ensure this study covers appropriate ages with regards to smoking uptake, we will focus on youths aged 11-15 years. We focus on smoking uptake within this age group because young people who establish a daily smoking habit by age 15 are less likely to quit or reduce their smoking as they move into adulthood (21,44–46). Smoking uptake in this study is defined as a series of transitions: initiation, representing initial trying; experimentation, or progression from initial trying to occasional use; escalation from occasional to daily use; and quitting (see Figure 2 (47)).

Using population representative longitudinal survey data, we will address the following research questions:

Q1. Has the implementation of the smoke-free public places legislation in the UK been associated with a step-change or change in the trend in the probability of youth smoking transitions?

For peer review only - http://bmjopen.bmj.com/site/about/guidelines.xhtml

Q2. Has the implementation of the smoke-free public places legislation in the UK been associated with a step-change or change in the trend in the inequalities in the probability of youth smoking transitions?

Q3 (a) Has the implementation of the smoke-free public places legislation in the UK been associated with a step-change or change in the trend in the prevalence of smoking among parents of UK youths? (b) Does the observed change (if any) differ by socioeconomic position?

Q4. (a) Do changes in parental smoking explain any impact of the smoke-free public places legislation on the probability of youth smoking transitions? (b) Has the implementation of the smoke-free public places legislation in the UK been associated with any change in the strength of the association between parental smoking and the probability of youth transitions? (c) Does the observed change (if any) differ by socioeconomic position?

Q5. How distinct are the impacts of the smoke-free public places legislation from the impacts of the change in the legal age for purchasing tobacco, the increasing prevalence of e-cigarettes, and the changes in tobacco taxation on youth smoking transition?

Methods and Analysis

Study design

The study will constitute secondary analyses of UK survey data (see below). The control group will be all UK youths aged 11-15 prior to the implementation of the smoking ban (in 2006 for youths living in Scotland and 2007 for those living elsewhere in the UK). The intervention group is all UK youths aged 11-15 after the implementation of the ban. The primary outcomes of interest are the rates of initiation, experimentation, escalation to daily smoking, and quitting within this age group. A secondary outcome of interest is the prevalence of smoking among parents of these youths. The causal interpretation of the differences between these groups rests on the assumption that the implementation of the smoke-free public places legislation is the only relevant difference between these groups, and that there are no other systematic differences between these groups that would account for their differences in outcomes.

Data

For peer review only - http://bmjopen.bmj.com/site/about/guidelines.xhtml

Data from the British Household Panel Survey Youth Sub-Sample and Understanding Society, both of which are freely available to *bona fide* researchers from the UK Data Archive under their normal terms and conditions (48,49). Both Understanding Society and BHPS studies obtained consent for data sharing from their participants. More information on the data sources, consent, and sampling can be found on BHPS and Understanding Society websites https://www.iser.essex.ac.uk/bhps and https://www.understandingsociety.ac.uk/ respectively. Data on youths' (11-15-year-olds) and parents' smoking history, and parents' socioeconomic position from the British Household Panel Survey (covering the period 1994 to 2009) will be merged with those of the recent Understanding Society Survey (from 2009 to 2016). The merger of these datasets gives coverage of the period from 1994 to 2016, with data from 2016 newly released in November 2017. As such, nine to ten years of post-legislation data will be available in the dataset. This amount of post-legislation data will be important in investigating the immediate as well as the long-term impact of the smoke-free public places legislation on youths smoking transition. Respondents will be included for each year they were aged 11-15 within this period (i.e. a maximum of five years of data per respondent).

Handling missing data

Missing data will be handled using multiple imputation, which ensures that all observed values in the set are retained (50,51). The multiple imputation will be conducted using an unconstrained two-level model in Mplus 8 (52), with person-years nested within persons.

Statistical analysis

Discrete-time event history analysis (53) will be conducted to examine whether changes in the probability of youth smoking transitions are associated with the implementation of the smoke-free public places legislation (Q1). The implementation of smoke-free legislation is viewed as a natural experiment (41,54), and analyses will examine if there is a step change or change in trend in the probability of smoking transitions after the country-specific implementation dates for the smoke-free legislation (i.e. 2006 for Scotland, 2007 for the rest of the UK). Youth will only be considered at risk for a transition once they have made the previous transition (i.e. they are only at risk for experimentation once they have tried smoking). Thus, data for each analysis will be right-censored at the year of the smoking transition (or age 15) and left censored prior to the previous transition (or age 11). Escalation

For peer review only - http://bmjopen.bmj.com/site/about/guidelines.xhtml

to daily smoking and quitting will be treated as two alternative outcomes, which young people will be at risk of once they commence occasional smoking. In line with previous work (47), youths who skipped smoking transition stages will be treated as making the intervening transitions in the same year. For instance, youths who transited from never having tried smoking to ex-smoker in the same year will be treated as having progressed to occasional smoker and having quit within the same year (but not as having escalated to daily smoking). Analyses will be adjusted for age, gender, the overall temporal trend in rates (which may encompass effects of other temporal increases in tobacco control), and overall differences in rates between UK countries.

We will then examine whether any effects observed on youth smoking transition differ by socioeconomic position (Q2). Parental education level will be used as an indicator of socioeconomic position. In cases where parental educational qualification for both parents differs, the higher qualification will be used. This will be added to the predictive models, alongside interactions with the variables representing the implementation of the legislation.

A multi-level logistic regression model (with person-years nested within persons) will be used to investigate whether there is a step-change or change in trend for the prevalence of parental smoking (any current smoking by either parent) after smoke-free public places legislations were implemented in the UK (Q3a); and whether observed changes (if any) differ by socioeconomic position (Q3b). This model will include all years of data for all respondents without any right or left censoring and will be adjusted for the young person's age, gender, the overall temporal trend in parental smoking, and overall differences between UK countries. The second stage of the modelling will additionally include parental education and interactions between parental education and the variables representing the implementation of the legislation.

Next, building on the models developed to assess whether the impact of the legislation on youth smoking transitions varied by socioeconomic position (Q2), parental smoking will also be included and we will assess whether this explains any observed differences associated with the implementation of the legislation (Q4a). We will test for interactions between parental smoking and the variables representing the implementation of the legislation to see if the influence of parental smoking changes after the legislation (Q4b), and then for a three-way interaction between parental smoking, legislation implementation and socioeconomic position to see if this varies by socioeconomic position (Q4c).

For peer review only - http://bmjopen.bmj.com/site/about/guidelines.xhtml

A final step will be to adjust the above models for relevant factors that may be associated with the implementation of the legislation. This will include annual levels of cigarette taxation, the change in the legal age of purchase for cigarettes from 16 to 18 in 2007 (that is, the implementation date differs from that of the smoke-free legislation by one year in Scotland only), and survey estimates of the annual prevalence of e-cigarettes from 2012 (55) (with e-cigarette prevalence coded as 0 prior to that date). As an additional sensitivity test, we will re-run analyses with the date of ban implementation transposed either 5 years forward or backward in time. If observed effects are genuinely attached to the ban rather than overall temporal trends, these transposed 'placebo' effect estimates should be weaker or null compared to those observed at the actual ban implementation dates.

Patient and Public Involvement

This study will not involve patients. The study will constitute secondary analyses of UK survey data. The dissemination of the results will include communication channels and public engagement events that will involve youths and parents.

Beneficiaries and target audiences

The project will be of interest to a range of academic and non-academic audiences. Research on tobacco control has a broad audience including epidemiologists, public health, policy researchers, governmental organisations, and the UK general public. The findings of this research will be of benefit to the governments of UK countries as they will provide information on important impacts of an existing policy. This information will be of use if the policy is ever considered for repeal or modification. The evidence on the mechanisms of impact on young people's smoking transition via their parents that will be generated from this project will be particularly valuable when considering extensions to smoke-free policies (for example, to more private spheres such as cars and homes, or to other public spaces such as outdoor parks) or other tobacco control policies which may have impacts on youth via parental smoking.

Another group who may benefit from this study are policymakers in other countries. Findings will be of international relevance as many countries are yet to implement partial or complete smoke-free public places legislation (56). The understanding of impacts and mechanisms gained from this study will help international policymakers evaluate whether similar effects could be gained by implementing smoke-free or other policies within their own countries.

For peer review only - http://bmjopen.bmj.com/site/about/guidelines.xhtml

Limitations

The causal interpretation of any impact on youth smoking uptake or parental smoking that we observe to be associated with the implementation of the smoke-free legislation rests on the assumption that this is the only relevant difference occurring at that point in time. Nevertheless, we will adjust for effects of some other relevant differences that could bias the results such as changes in tobacco taxation and the increasing availability of e-cigarettes, to see if we can differentiate the effects of the smoke-free legislation. The change in the legal age for purchase of cigarettes from age 16 to 18 in 2007 is particularly problematic, as it coincides so closely with the timing of the implementation of the smoke-free legislation (only differing by a year in Scotland, and being concurrent in the rest of the UK), so it may be difficult to statistically distinguish their effects. At worst this means that findings could be interpreted in terms of the package of policies implemented at that point (i.e. the smoke-free legislation and the change in legal age), but our investigation of mechanisms of impact via parental smoking remains relevant in this regard. If some of the impacts can be attributed to parental smoking, this will further strengthen the case for a causal effect of the smoke-free legislation, as the change in legal age could be expected to have much less of an impact on adult smokers.

Discussion

Smoke-free public places legislation has already been the subject of many evaluative studies and is widely viewed as a success, particularly in reducing exposure to second-hand smoke; but there remain gaps in the evidence base for this policy. This project would fill one of the most important of these gaps, relating to a crucial population group in tobacco control, i.e. youth. If the project shows positive impacts of the smoke-free legislation, then it will add to the evidence supporting this policy. If the project shows negative impacts of the smoking ban -such as the widening of inequalities in youth smoking uptake-, then it provides important information that should be weighed against the other benefits already shown in previous research; and may point to other measures that might be taken in combination with smoke-free legislation for maximum benefit.

The difficulty in establishing whether observed associations between events are causal is a common problem in the field of epidemiology (57), but our natural experimental approach will help strengthen the case for a causal effect. Furthermore, the interest of evaluators of

For peer review only - http://bmjopen.bmj.com/site/about/guidelines.xhtml

social policies or interventions goes beyond overall effects. An understanding of the mechanisms by which an effect occurs is crucial to social policy evaluators. Such understanding can contribute to establishing the transferability of such policies to different contexts while achieving similar effects. This project will achieve this grounded understanding by explicating the role of parental smoking in any impact on youth smoking transition. Similarly, the fine definition of smoking transition which includes all stages of smoking (from initiation to escalation or quitting) will help elucidate which specific stages of the process of smoking transition are affected by the smoke-free public places legislation, and to what extent the effect is.

Finally, e-cigarettes are the focus of current controversy in the field of tobacco control. Many advocate e-cigarettes for the potential harm-reduction which could be achieved if smokers switched from tobacco to e-cigarettes, but there are also concerns that they may re-normalise smoking behaviours (58), and put current successes in tobacco control at risk. A particular concern is that e-cigarettes could help establish nicotine addiction in young people and lead to increases in cigarette smoking (59), though there is little evidence of this as yet (43). By differentiating effects of the smoke-free public places legislation from those of increasing e-cigarette prevalence, we will generate evidence about the independent effects of each on youth smoking transitions, making an important contribution to these current policy debates.

Ethical and dissemination

This study will utilise secondary data that are anonymised and obtained from studies that have already undergone ethical review. The Understanding Society study protocol was reviewed and approved by the Ethics Committee of the University of Essex; while the BHPS complied with the ethical guidelines of the Social Research Association.

The dissemination of the study findings will adopt multiple communication channels targeting both scientific and non-scientific audiences. The key communication channels will include peer-reviewed journal articles, conference presentations, press releases coinciding with publications, online blogs, and public engagement events. Also, policymakers and stakeholders will be updated on the progress of this study and on preliminary findings via a virtual stakeholder network.

For peer review only - http://bmjopen.bmj.com/site/about/guidelines.xhtml

References

1. Ali R, Hay S. Smoking prevalence and attributable disease burden in 195 countries and territories, 1990–2015: a systematic analysis from the Global Burden of Disease Study 2015. 2017;

2. Forouzanfar MH, Afshin A, Alexander LT, Aasvang GM, Bjertness E, Htet AS, et al. Global, regional, and national comparative risk assessment of 79 behavioural, environmental and occupational, and metabolic risks or clusters of risks, 1990-2015: a systematic analysis for the Global Burden of Disease Study 2015. 2016;

3. Brown T, Platt S, Amos A. Equity impact of interventions and policies to reduce smoking in youth: systematic review. Tob Control. 2014;tobaccocontrol–2013.

4. Hiscock R, Bauld L, Amos A, Fidler JA, Munafò M. Socioeconomic status and smoking: a review. Ann N Y Acad Sci. 2012;1248(1):107–123.

5. Barreto SM, de Figueiredo RC, Giatti L. Socioeconomic inequalities in youth smoking in Brazil. BMJ Open. 2013;3(12):e003538.

6. Global and Public Health/Population Health/HB/ cost centre. Towards a smoke-free generation: tobacco control plan for England - GOV.UK [Internet]. 2017 [cited 2017 Oct 25]. Available from: https://www.gov.uk/government/publications/towards-a-smoke-free-generation-tobacco-control-plan-for-england

7. The Scottish Government. Creating a Tobacco-Free Generation: A Tobacco Control Strategy for Scotland - Google Search [Internet]. The Scottish Government, Edinburgh; 2013 [cited 2017 Oct 25]. Available from: http://www.gov.scot/resource/0041/00417331.pdf

8. Frazer K, Callinan JE, McHugh J, van Baarsel S, Clarke A, Doherty K, et al. Legislative smoking bans for reducing harms from secondhand smoke exposure, smoking prevalence and tobacco consumption. Cochrane Libr [Internet]. 2016 [cited 2017 Sep 18]; Available from: http://onlinelibrary.wiley.com/doi/10.1002/14651858.CD005992.pub3/full

9. Mackay D, Haw S, Ayres JG, Fischbacher C, Pell JP. Smoke-free legislation and hospitalizations for childhood asthma. N Engl J Med. 2010;363(12):1139–1145.

10. Pell JP, Haw S, Cobbe S, Newby DE, Pell AC, Fischbacher C, et al. Smoke-free legislation and hospitalizations for acute coronary syndrome. N Engl J Med. 2008;359(5):482–491.

11. Mackay DF, Nelson SM, Haw SJ, Pell JP. Impact of Scotland's smoke-free legislation on pregnancy complications: retrospective cohort study. PLoS Med. 2012;9(3):e1001175.

12. Been JV, Nurmatov UB, Cox B, Nawrot TS, van Schayck CP, Sheikh A. Effect of smoke-free legislation on perinatal and child health: a systematic review and meta-analysis. The Lancet. 2014;383(9928):1549–1560.

For peer review only - http://bmjopen.bmj.com/site/about/guidelines.xhtml

13. Faber T, Kumar A, Mackenbach JP, Millett C, Basu S, Sheikh A, et al. Effect of tobacco control policies on perinatal and child health: a systematic review and meta-analysis. Lancet Public Health. 2017;2(9):e420–e437.

14. Katikireddi SV, Der G, Roberts C, Haw S. Has childhood smoking reduced following smoke-free public places legislation? A segmented regression analysis of cross-sectional UK school-based surveys. Nicotine Tob Res. 2016;18(7):1670–1674.

15. Britton J. Smoke-free policy and child health. Lancet Public Health. 2017;2(9):e392–e393.

16. Ritchie D, Amos A, Martin C. Public places after smoke-free—A qualitative exploration of the changes in smoking behaviour. Health Place. 2010 May 1;16(3):461–9.

17. Haw SJ, Gruer L, Amos A, Currie C, Fischbacher C, Fong GT, et al. Legislation on smoking in enclosed public places in Scotland: how will we evaluate the impact? J Public Health. 2006;28(1):24–30.

18. Hamilton WL, Biener L, Brennan RT. Do local tobacco regulations influence perceived smoking norms? Evidence from adult and youth surveys in Massachusetts. Health Educ Res. 2007;23(4):709–722.

19. Lenk KM, Toomey TL, Shi Q, Erickson DJ, Forster JL. Do sources of cigarettes among adolescents vary by age over time? J Child Adolesc Subst Abuse. 2014;23(2):137–143.

20. Jansen P, Toomey TL, Nelson TF, Fabian LE, Lenk KM, Forster JL. Sources of cigarettes among adolescent smokers: Free or purchased? Am J Health Educ. 2011;42(3):154–160.

21. Tyas SL, Pederson LL. Psychosocial factors related to adolescent smoking: a critical review of the literature. Tob Control. 1998;7(4):409–420.

22. Jones AM, Laporte A, Rice N, Zucchelli E. Do public smoking bans have an impact on active smoking? Evidence from the UK. Health Econ. 2015;24(2):175–192.

23. Monson E, Arsenault N. Effects of enactment of legislative (public) smoking bans on voluntary home smoking restrictions: A review. Nicotine Tob Res. 2017;19(2):141–148.

24. House of Commons - Health - Minutes of Evidence. Examination of witnesses (questions 1–19) 23rd Feb 2005 [Internet]. 2005 [cited 2017 Sep 18]. Available from: https://publications.parliament.uk/pa/cm200405/cmselect/cmhealth/358/5022302.htm

25. López MJ, Nebot M, Schiaffino A, Pérez-Ríos M, Fu M, Ariza C, et al. Two-year impact of the Spanish smoking law on exposure to secondhand smoke: evidence of the failure of the 'Spanish model.' Tob Control. 2011;tc–2010.

26. Adda J, Cornaglia F. The effect of bans and taxes on passive smoking. Am Econ J Appl Econ. 2010;2(1):1–32.

27. Lee JT, Glantz SA, Millett C. Effect of smoke-free legislation on adult smoking behaviour in England in the 18 months following implementation. PloS One. 2011;6(6):e20933.

For peer review only - http://bmjopen.bmj.com/site/about/guidelines.xhtml

28. Carpenter C, Postolek S, Warman C. Public-place smoking laws and exposure to environmental tobacco smoke (ETS). Am Econ J Econ Policy. 2011;3(3):35–61.

29. Mons U, Nagelhout GE, Allwright S, Guignard R, van den Putte B, Willemsen MC, et al. Impact of national smoke-free legislation on home smoking bans: findings from the International Tobacco Control Policy Evaluation Project Europe Surveys. Tob Control. 2012;tobaccocontrol–2011.

30. Martínez-Sánchez JM, Blanch C, Fu M, Gallus S, La Vecchia C, Fernández E. Do smoke-free policies in work and public places increase smoking in private venues? Tob Control. 2013;tobaccocontrol–2012.

31. Ferketich AK, Lugo A, La Vecchia C, Fernandez E, Boffetta P, Clancy L, et al. Relation between national-level tobacco control policies and individual-level voluntary home smoking bans in Europe. Tob Control. 2016;25(1):60–65.

32. Hyland A, Hassan LM, Higbee C, Boudreau C, Fong GT, Borland R, et al. The impact of smokefree legislation in Scotland: results from the Scottish ITC Scotland/UK longitudinal surveys. Eur J Public Health. 2009;19(2):198–205.

33. Holliday JC, Moore GF, Moore LA. Changes in child exposure to secondhand smoke after implementation of smoke-free legislation in Wales: a repeated cross-sectional study. BMC Public Health. 2009;9(1):430.

34. Akhtar PC, Currie DB, Currie CE, Haw SJ. Changes in child exposure to environmental tobacco smoke (CHETS) study after implementation of smoke-free legislation in Scotland: national cross sectional survey. Bmj. 2007;335(7619):545.

35. Sims M, Bauld L, Gilmore A. England's legislation on smoking in indoor public places and work-places: impact on the most exposed children. Addiction. 2012;107(11):2009–2016.

36. Jarvis MJ, Sims M, Gilmore A, Mindell J. Impact of smoke-free legislation on children's exposure to secondhand smoke: cotinine data from the Health Survey for England. Tob Control. 2011;tc–2010.

37. Lidón-Moyano C, Martínez-Sánchez JM, Fu M, Ballbè M, Martín-Sánchez JC, Martínez C, et al. Secondhand smoke risk perception and smoke-free rules in homes: a cross-sectional study in Barcelona (Spain). BMJ Open. 2017;7(1):e014207.

38. Klein EG, Forster JL, Erickson DJ, Lytle LA, Schillo B. The relationship between local clean indoor air policies and smoking behaviours in Minnesota youth. Tob Control. 2009 Apr 1;18(2):132–7.

39. Moore GF, Currie D, Gilmore G, Holliday JC, Moore L. Socioeconomic inequalities in childhood exposure to secondhand smoke before and after smoke-free legislation in three UK countries. J Public Health. 2012;34(4):599–608.

40. Akhtar PC, Haw SJ, Levin KA, Currie DB, Zachary R, Currie CE. Socioeconomic differences in second-hand smoke exposure among children in Scotland after introduction of the smoke-free legislation. J Epidemiol Community Health. 2010;64(4):341–346.

For peer review only - http://bmjopen.bmj.com/site/about/guidelines.xhtml

41. Craig P, Katikireddi SV, Leyland A, Popham F. Natural Experiments: An Overview of Methods, Approaches, and Contributions to Public Health Intervention Research. Annu Rev Public Health. 2017;38:39–56.

42. Sims M, Salway R, Langley T, Lewis S, McNeill A, Szatkowski L, et al. Effectiveness of tobacco control television advertising in changing tobacco use in England: a population-based cross-sectional study. Addiction. 2014;109(6):986–994.

43. McNeill A, Brose LS, Calder R, Hitchman SC, Hajek P, McRobbie H. E-cigarettes: an evidence update. Public Health Engl. 2015;3.

44. Chassin L, Presson CC, Pitts SC, Sherman SJ. The natural history of cigarette smoking from adolescence to adulthood in a midwestern community sample: multiple trajectories and their psychosocial correlates. Health Psychol. 2000;19(3):223.

45. Kandel DB, Logan JA. Patterns of drug use from adolescence to young adulthood: I. Periods of risk for initiation, continued use, and discontinuation. Am J Public Health. 1984;74(7):660–666.

46. Wellman RJ, DiFranza JR, Savageau JA, Dussault GF. Short term patterns of early smoking acquisition. Tob Control. 2004;13(3):251–257.

47. Green MJ, Leyland AH, Sweeting H, Benzeval M. Socioeconomic position and early adolescent smoking development: evidence from the British Youth Panel Survey (1994–2008). Tob Control. 2016;25(2):203–210.

48. Taylor M, Change ERC on M-S. British Household Panel Survey user manual vol. A: Introduction, technical report and appendices. ESRC Research Centre on Micro-Social Change; 1995.

49. Knies G. User guide – Understanding Society: The UK Household Longitudinal Study waves 1-6 [Internet]. 2016 [cited 2017 Nov 16]. Available from: https://www.understandingsociety.ac.uk/documentation/mainstage

50. Sterne JA, White IR, Carlin JB, Spratt M, Royston P, Kenward MG, et al. Multiple imputation for missing data in epidemiological and clinical research: potential and pitfalls. Bmj. 2009;338:b2393.

51. Allison PD. Missing data: Quantitative applications in the social sciences. Br J Math Stat Psychol. 2002;55(1):193–196.

52. Muthén LK, Muthén BO. Mplus User's Guide. Los Angeles, CA: Muthen & Muthen; 1998-2010. CzeglédiE Body Dissatisfaction Trait Anxiety Self-Esteem Young Men. 2016;

53. Allison PD. Discrete-Time Methods for the Analysis of Event Histories. Sociol Methodol. 1982;13:61–98.

54. Craig P, Cooper C, Gunnell D, Haw S, Lawson K, Macintyre S, et al. Using natural experiments to evaluate population health interventions: new Medical Research Council guidance. J Epidemiol Community Health. 2012;jech–2011.

For peer review only - http://bmjopen.bmj.com/site/about/guidelines.xhtml

55. Action on Smoking and Health. Use of electronic cigarettes (vapourisers) among adults in Great Britain [Internet]. 2016 [cited 2017 Nov 22]. Available from: http://colinmendelsohn.com.au/files/2814/9446/2312/ASH._Use_of_electronic_cigarettes_vapourisers_among_adults_in_Great_Britain._Fact_sheet._May_2017.pdf

56. World Health Organization. WHO REPORT ON THE GLOBAL TOBACCO EPIDEMIC, 2017: Monitoring tobacco use and prevention policies [Internet]. Geneva: WHO; 2017. Available from: http://apps.who.int/iris/bitstream/10665/255874/1/9789241512824-eng.pdf?ua=1&ua=1

57. Goodman S, Samet J. In Schottenfeld, D., and Fraumeni, J. F., (2006) Cancer Epidemiology and Prevention. Oxford University Press; 2006. 1411 p.

58. Fairchild AL, Bayer R, Colgrove J. The renormalization of smoking? E-cigarettes and the tobacco "endgame." N Engl J Med. 2014;370(4):293–295.

59. Hilton S, Weishaar H, Sweeting H, Trevisan F, Katikireddi SV. E-cigarettes, a safer alternative for teenagers? A UK focus group study of teenagers' views. BMJ Open. 2016;6(11):e013271.

Authors' Contributions:

MG, PC and SVK contributed to the conception and design of the study. All authors contributed to developing the analytical methods. The manuscript was drafted by PEA and further revised by MG, PC, SVK and PEA. All authors approved the publication of the protocol.

Funding statement

This study is funded by the Economics & Social Research Council Secondary Data Analysis Initiative (300405-01). All authors receive funding from the Medical Research Council (MC_UU_12017/13 & MC_UU_12017/15) and Scottish Government Chief Scientist Office (SPSHU13 & SPHSU15). SVK is funded by an NRS Senior Clinical Fellowship (SCAF/15/02).

Competing interest

All authors declare no conflicts of interest.

Figure Legends

Figure 1: Conceptual framework for the impact of smoke-free public places legislation on youth smoking transition.

For peer review only - http://bmjopen.bmj.com/site/about/guidelines.xhtml

Figure 2: Smoking stages for early adolescents adapted from ‘Socioeconomic position and early adolescent smoking development: evidence from the British Youth Panel Survey (1994–2008)’ by Green MJ, Leyland AH, Sweeting H, and Benzeval M., 2016, Tobacco Control, 25(2):203–210.

For peer review only

For peer review only - http://bmjopen.bmj.com/site/about/guidelines.xhtml

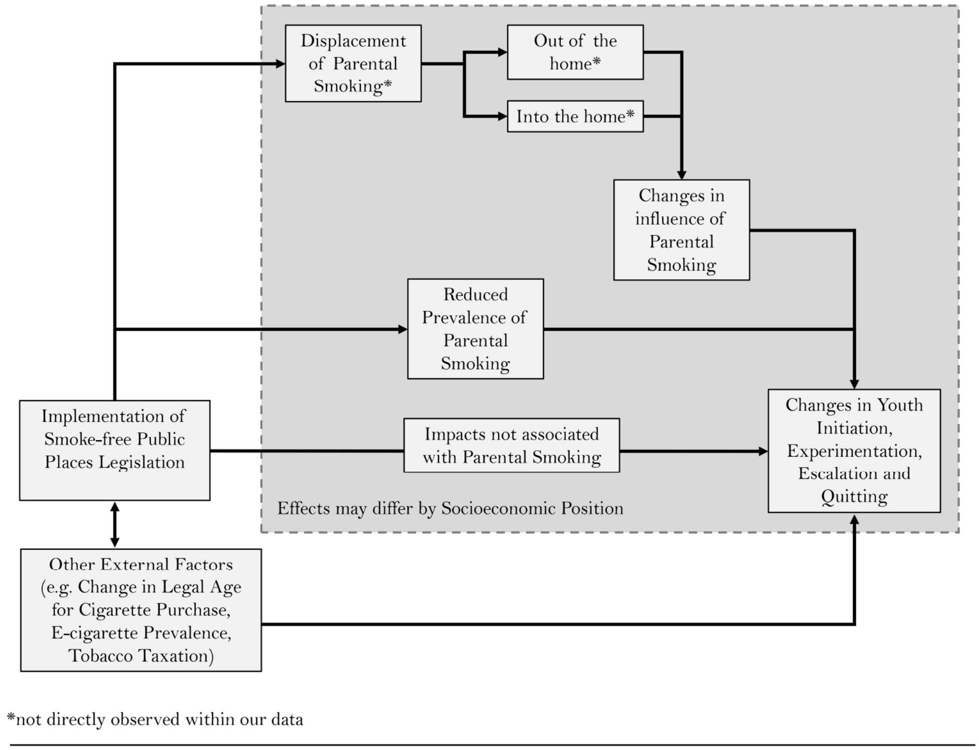

Conceptual framework for the impact of smoke-free public places legislation on youth smoking transition

209x163mm (300 x 300 DPI)

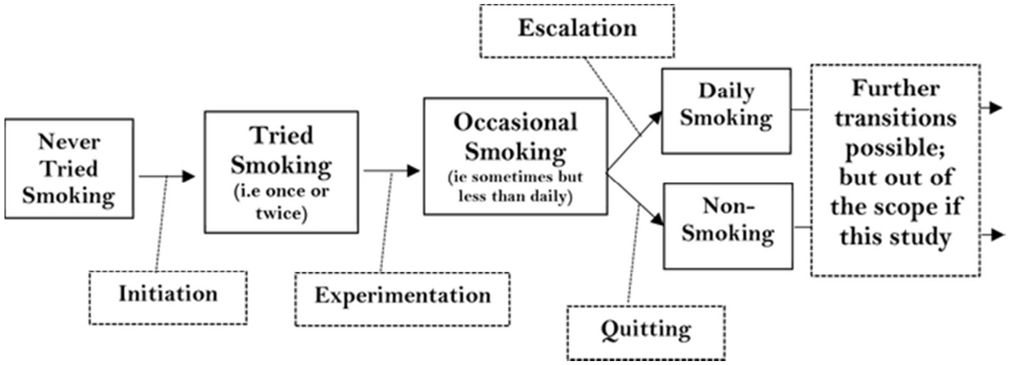

Smoking stages for early adolescents adapted from 'Socioeconomic position and early adolescent smoking development: evidence from the British Youth Panel Survey (1994–2008)' by Green MJ, Leyland AH, Sweeting H, and Benzeval M., 2016, Tobacco Control, 25(2):203–210

57x20mm (300 x 300 DPI)
