# Supplementary material for: Impacts of smoke-free public places legislation on inequalities in youth smoking uptake: study protocol for a secondary analysis of UK survey data
Source: BMJ Open. 2018 Mar 27;8(3):e022490. doi: 10.1136/bmjopen-2018-022490 (PMC5875608; doi:10.1136/bmjopen-2018-022490)
Supplement: Reviewer comments [file bmjopen-2018-022490.reviewer_comments.pdf]

PEER REVIEW HISTORY

BMJ Open publishes all reviews undertaken for accepted manuscripts. Reviewers are asked to complete a checklist review form (**http://bmjopen.bmj.com/site/about/resources/checklist.pdf**) and are provided with free text boxes to elaborate on their assessment. These free text comments are reproduced below.

ARTICLE DETAILS

**TITLE (PROVISIONAL)**	IMPACTS OF SMOKE-FREE PUBLIC PLACES LEGISLATION ON INEQUALITIES IN YOUTH SMOKING UPTAKE: STUDY PROTOCOL
**AUTHORS**	Anyanwu, Philip; Craig, Peter; Katikireddi, Srinivasa; Green, Michael

This article was not externally reviewed at BMJ Open. Protocols that have been independently assessed prior to submission to BMJ Open are usually fast-tracked to publication on the grounds that further substantial changes will not be possible. This independent assessment will usually be external, independent review for both a substantial grant award from a non-commercial or government funder and ethics approval.
